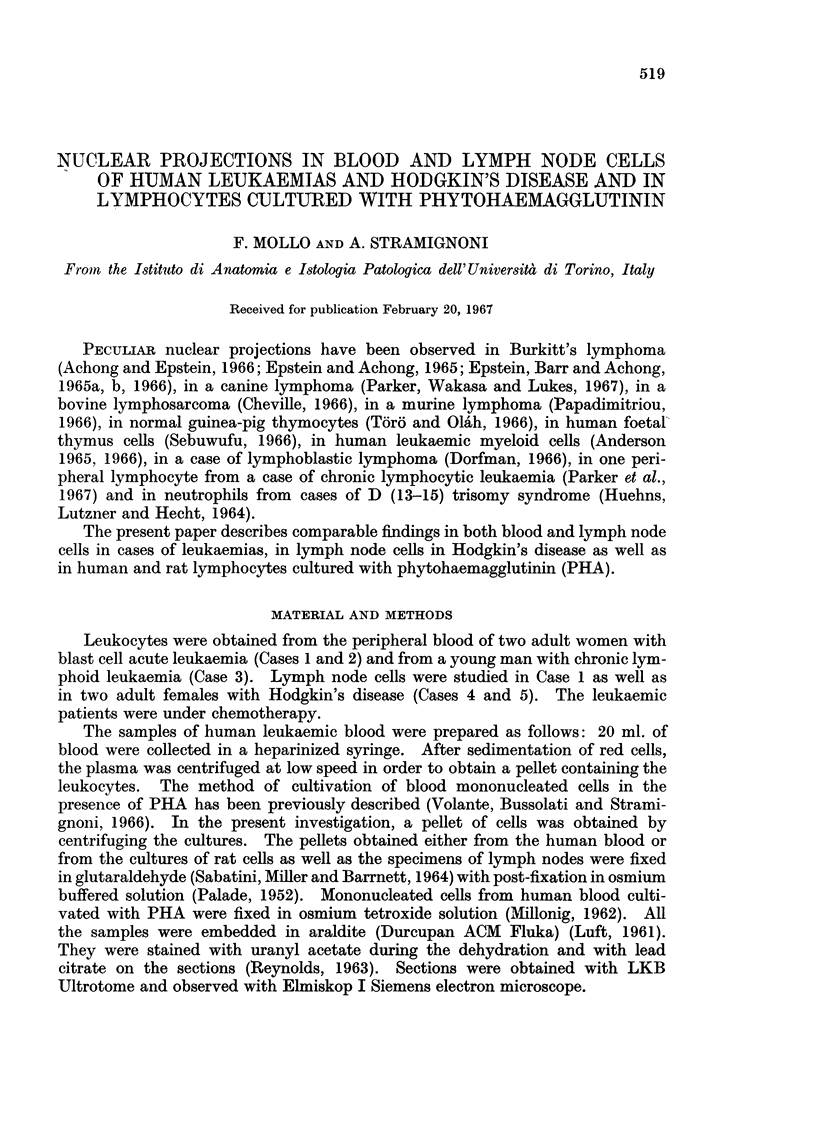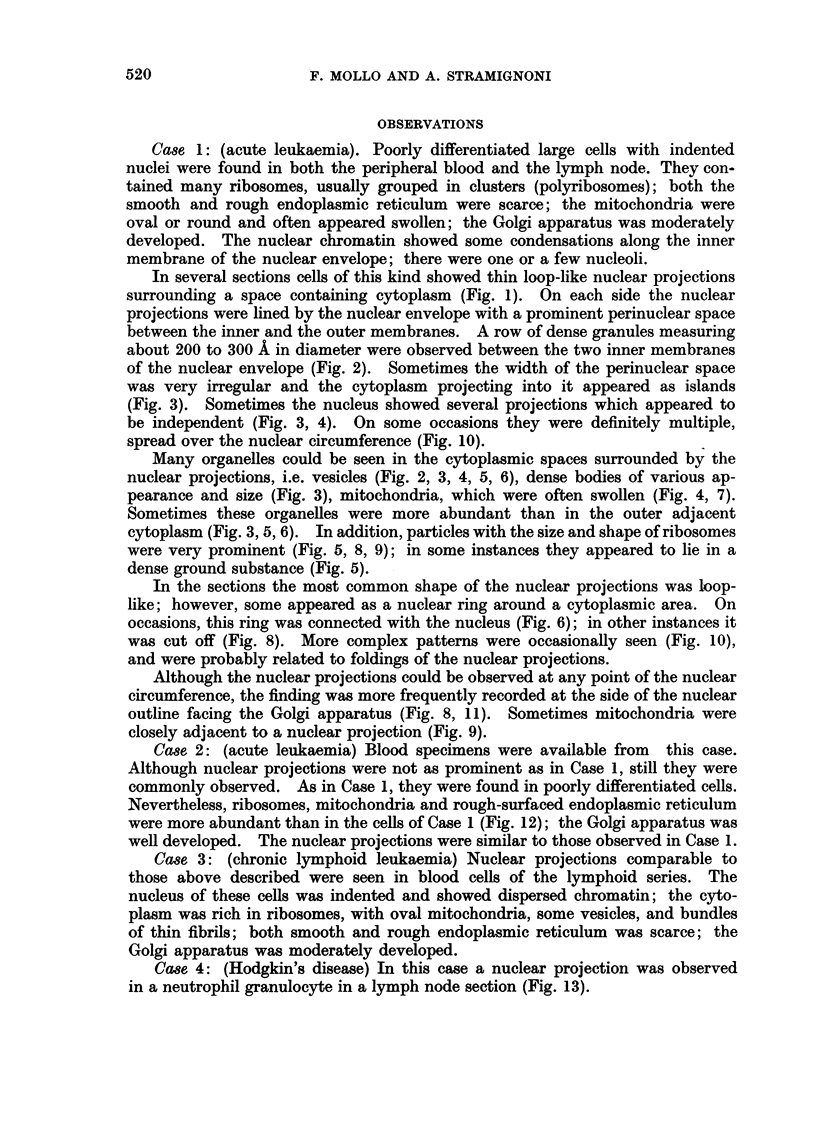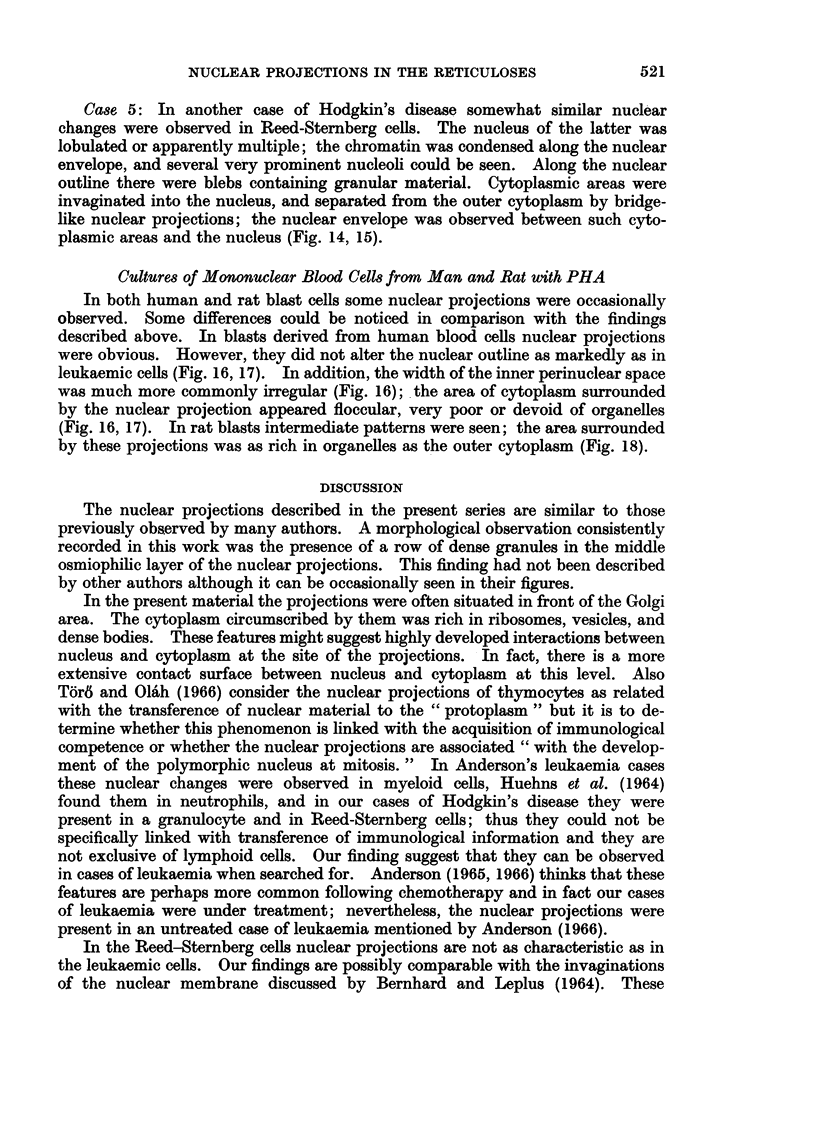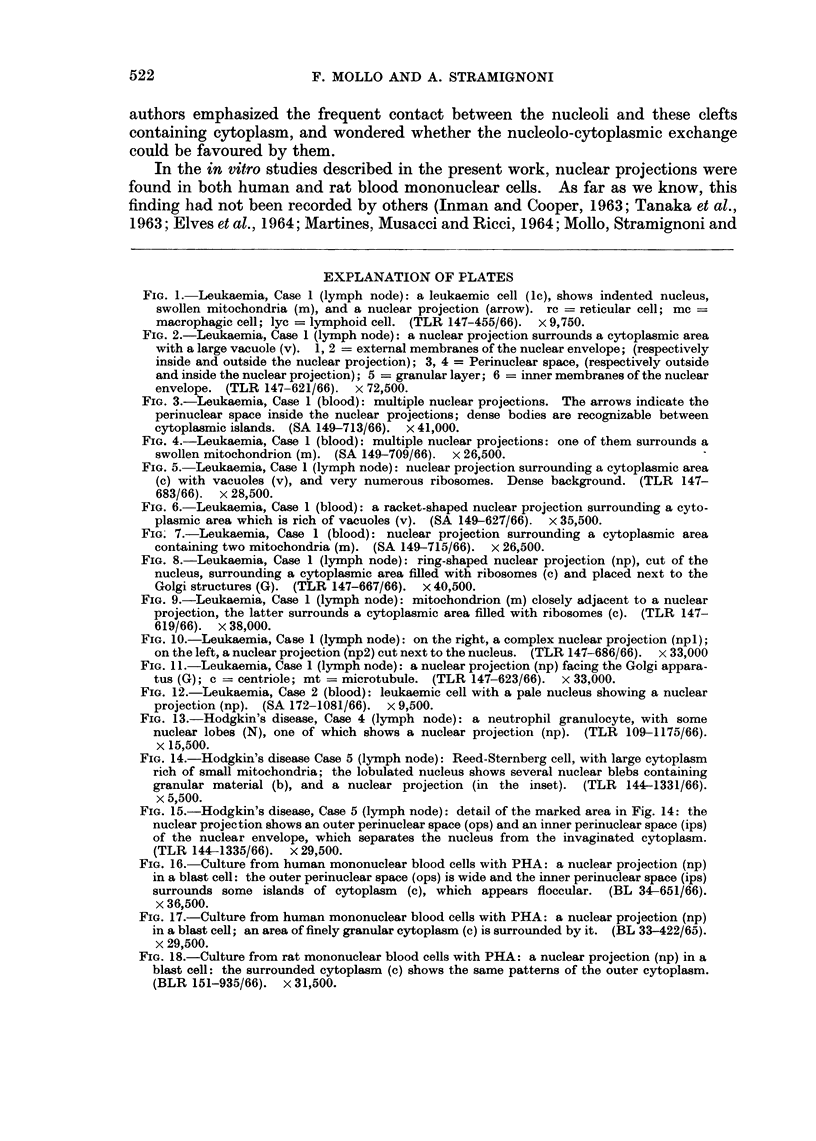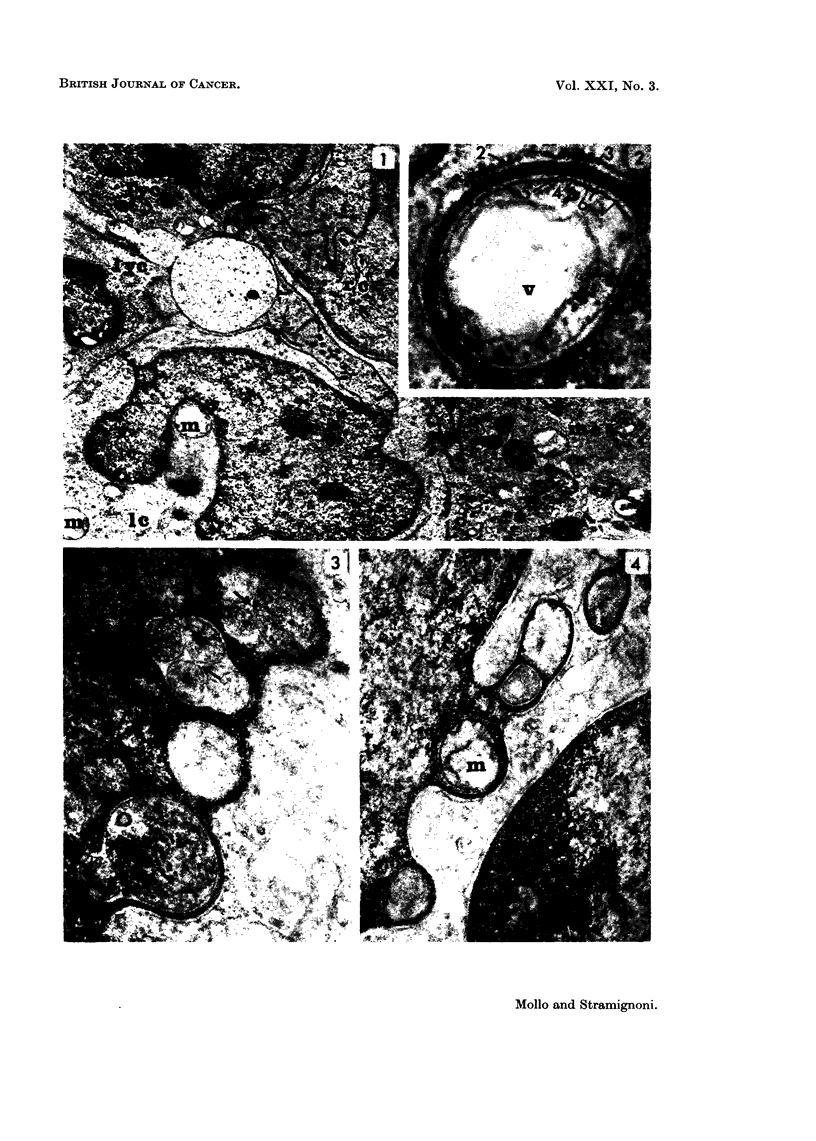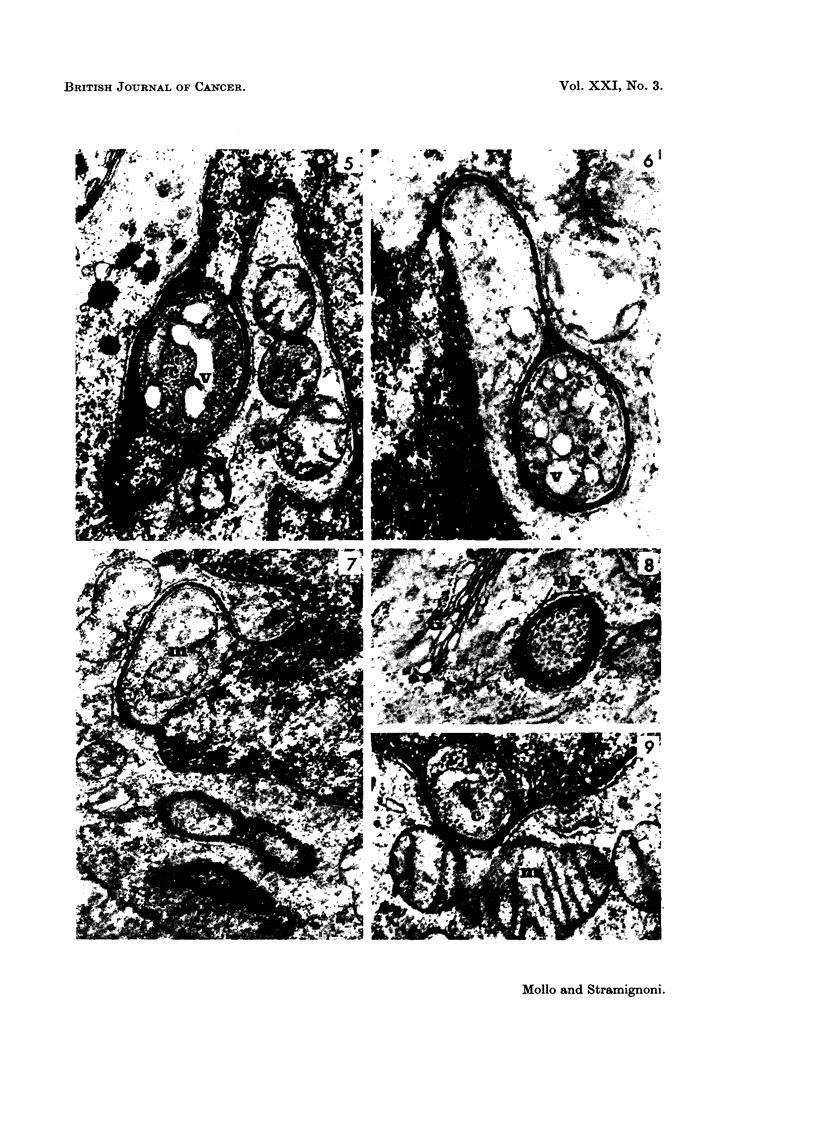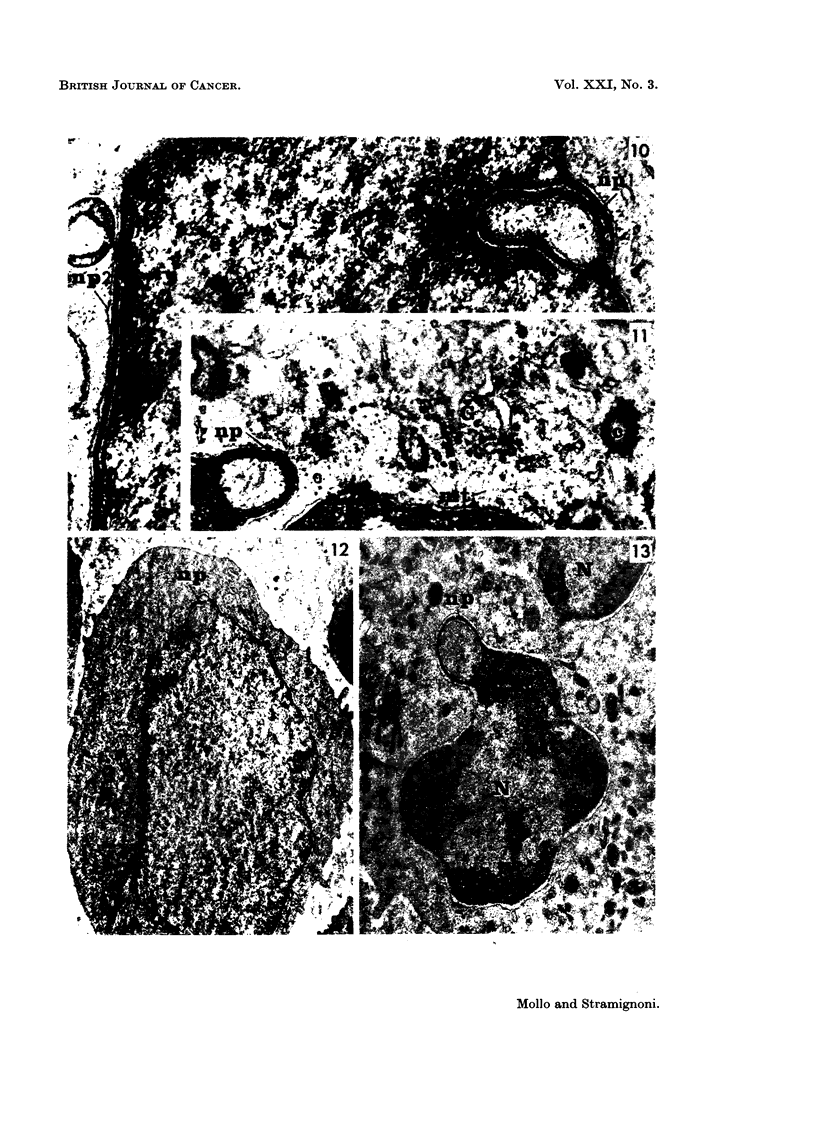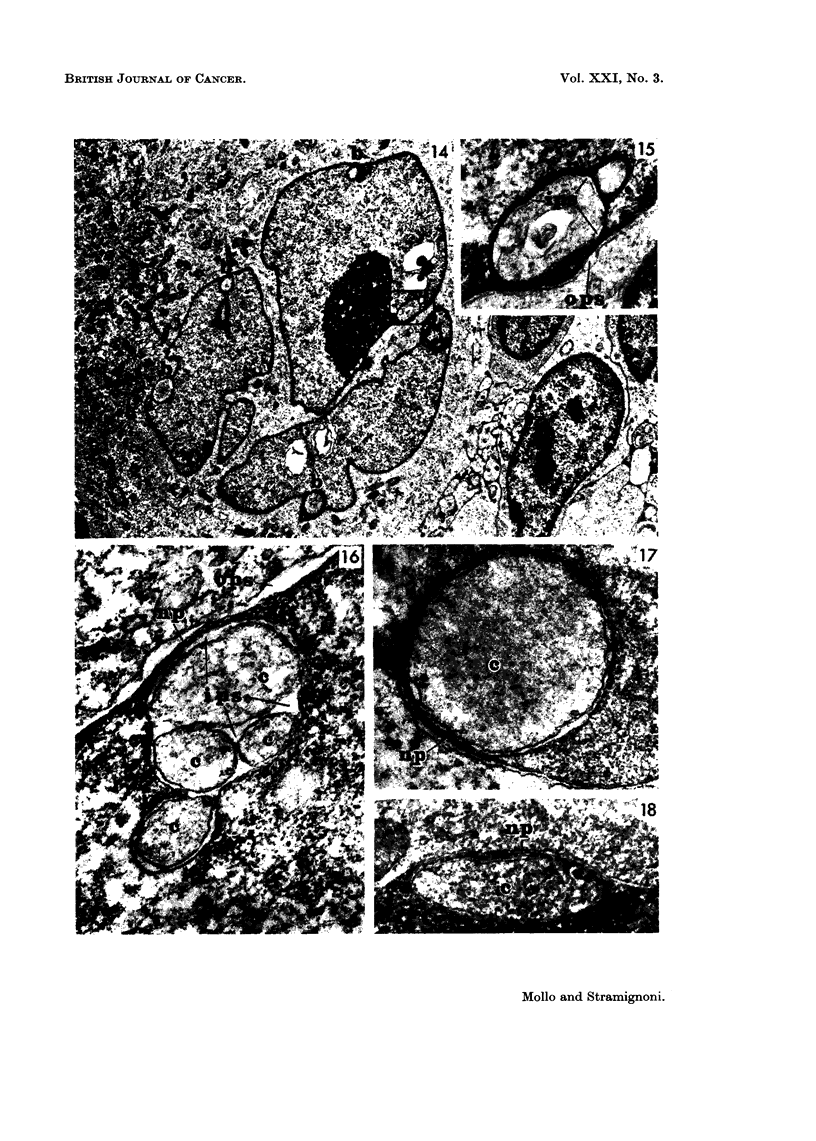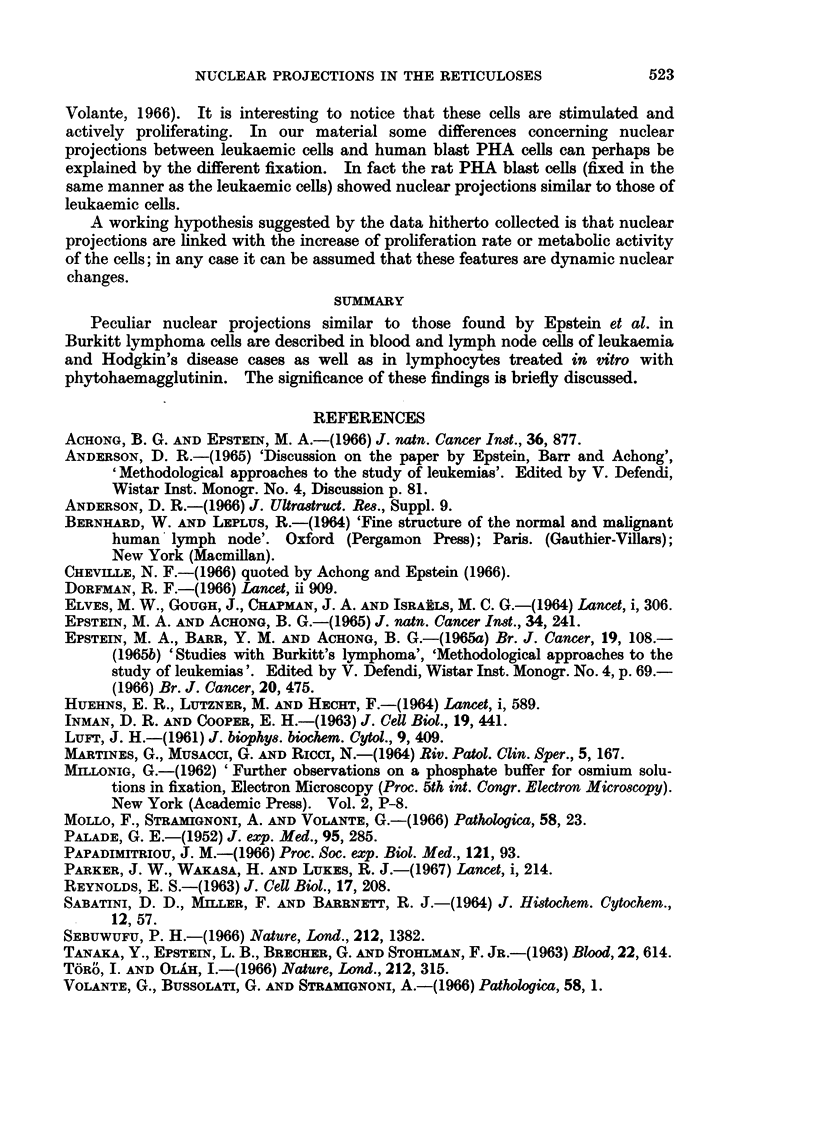# Nuclear projections in blood and lymph node cells of human leukaemias and Hodgkin's disease and in lymphocytes cultured with phytohaemagglutinin.

**DOI:** 10.1038/bjc.1967.61

**Published:** 1967-09

**Authors:** F. Mollo, A. Stramignoni

## Abstract

**Images:**


### NUCLEAR PROJECTIONS IN BLOOD AND LYMPH NODE CELLS OF HUMAN LEUKAEMIAS AND HODGKIN'S DISEASE AND IN LYMPHOCYTES CULTURED WITH PHYTOHAEMAGGLUTININ

F. MOLLO AND A. STRAMIGNONI

*From the Istituto di Anatomia e Istologia Patologica dell'Università di Torino, Italy*

Received for publication February 20, 1967

PECULIAR nuclear projections have been observed in Burkitt's lymphoma (Achong and Epstein, 1966; Epstein and Achong, 1965; Epstein, Barr and Achong, 1965a, b, 1966), in a canine lymphoma (Parker, Wakasa and Lukes, 1967), in a bovine lymphosarcoma (Cheville, 1966), in a murine lymphoma (Papadimitriou, 1966), in normal guinea-pig thymocytes (Törö and Oláh, 1966), in human foetal thymus cells (Sebuwufu, 1966), in human leukaemic myeloid cells (Anderson 1965, 1966), in a case of lymphoblastic lymphoma (Dorfman, 1966), in one peripheral lymphocyte from a case of chronic lymphocytic leukaemia (Parker *et al.*, 1967) and in neutrophils from cases of D (13–15) trisomy syndrome (Huehns, Lutzner and Hecht, 1964).

The present paper describes comparable findings in both blood and lymph node cells in cases of leukaemias, in lymph node cells in Hodgkin's disease as well as in human and rat lymphocytes cultured with phytohaemagglutinin (PHA).

### MATERIAL AND METHODS

Leukocytes were obtained from the peripheral blood of two adult women with blast cell acute leukaemia (Cases 1 and 2) and from a young man with chronic lymphoid leukaemia (Case 3). Lymph node cells were studied in Case 1 as well as in two adult females with Hodgkin's disease (Cases 4 and 5). The leukaemic patients were under chemotherapy.

The samples of human leukaemic blood were prepared as follows: 20 ml. of blood were collected in a heparinized syringe. After sedimentation of red cells, the plasma was centrifuged at low speed in order to obtain a pellet containing the leukocytes. The method of cultivation of blood mononucleated cells in the presence of PHA has been previously described (Volante, Bussolati and Stramignoni, 1966). In the present investigation, a pellet of cells was obtained by centrifuging the cultures. The pellets obtained either from the human blood or from the cultures of rat cells as well as the specimens of lymph nodes were fixed in glutaraldehyde (Sabatini, Miller and Barrnett, 1964) with post-fixation in osmium buffered solution (Palade, 1952). Mononucleated cells from human blood cultivated with PHA were fixed in osmium tetroxide solution (Millonig, 1962). All the samples were embedded in araldite (Durcupan ACM Fluka) (Luft, 1961). They were stained with uranyl acetate during the dehydration and with lead citrate on the sections (Reynolds, 1963). Sections were obtained with LKB Ultrotome and observed with Elmiskop I Siemens electron microscope.

### OBSERVATIONS

*Case* 1: (acute leukaemia). Poorly differentiated large cells with indented nuclei were found in both the peripheral blood and the lymph node. They contained many ribosomes, usually grouped in clusters (polyribosomes); both the smooth and rough endoplasmic reticulum were scarce; the mitochondria were oval or round and often appeared swollen; the Golgi apparatus was moderately developed. The nuclear chromatin showed some condensations along the inner membrane of the nuclear envelope; there were one or a few nucleoli.

In several sections cells of this kind showed thin loop-like nuclear projections surrounding a space containing cytoplasm (Fig. 1). On each side the nuclear projections were lined by the nuclear envelope with a prominent perinuclear space between the inner and the outer membranes. A row of dense granules measuring about 200 to 300 Å in diameter were observed between the two inner membranes of the nuclear envelope (Fig. 2). Sometimes the width of the perinuclear space was very irregular and the cytoplasm projecting into it appeared as islands (Fig. 3). Sometimes the nucleus showed several projections which appeared to be independent (Fig. 3, 4). On some occasions they were definitely multiple, spread over the nuclear circumference (Fig. 10).

Many organelles could be seen in the cytoplasmic spaces surrounded by the nuclear projections, i.e. vesicles (Fig. 2, 3, 4, 5, 6), dense bodies of various appearance and size (Fig. 3), mitochondria, which were often swollen (Fig. 4, 7). Sometimes these organelles were more abundant than in the outer adjacent cytoplasm (Fig. 3, 5, 6). In addition, particles with the size and shape of ribosomes were very prominent (Fig. 5, 8, 9); in some instances they appeared to lie in a dense ground substance (Fig. 5).

In the sections the most common shape of the nuclear projections was loop-like; however, some appeared as a nuclear ring around a cytoplasmic area. On occasions, this ring was connected with the nucleus (Fig. 6); in other instances it was cut off (Fig. 8). More complex patterns were occasionally seen (Fig. 10), and were probably related to foldings of the nuclear projections.

Although the nuclear projections could be observed at any point of the nuclear circumference, the finding was more frequently recorded at the side of the nuclear outline facing the Golgi apparatus (Fig. 8, 11). Sometimes mitochondria were closely adjacent to a nuclear projection (Fig. 9).

*Case* 2: (acute leukaemia) Blood specimens were available from this case. Although nuclear projections were not as prominent as in Case 1, still they were commonly observed. As in Case 1, they were found in poorly differentiated cells. Nevertheless, ribosomes, mitochondria and rough-surfaced endoplasmic reticulum were more abundant than in the cells of Case 1 (Fig. 12); the Golgi apparatus was well developed. The nuclear projections were similar to those observed in Case 1.

*Case* 3: (chronic lymphoid leukaemia) Nuclear projections comparable to those above described were seen in blood cells of the lymphoid series. The nucleus of these cells was indented and showed dispersed chromatin; the cytoplasm was rich in ribosomes, with oval mitochondria, some vesicles, and bundles of thin fibrils; both smooth and rough endoplasmic reticulum was scarce; the Golgi apparatus was moderately developed.

*Case* 4: (Hodgkin's disease) In this case a nuclear projection was observed in a neutrophil granulocyte in a lymph node section (Fig. 13).

*Case* 5: In another case of Hodgkin's disease somewhat similar nuclear changes were observed in Reed-Sternberg cells. The nucleus of the latter was lobulated or apparently multiple; the chromatin was condensed along the nuclear envelope, and several very prominent nucleoli could be seen. Along the nuclear outline there were blebs containing granular material. Cytoplasmic areas were invaginated into the nucleus, and separated from the outer cytoplasm by bridge-like nuclear projections; the nuclear envelope was observed between such cytoplasmic areas and the nucleus (Fig. 14, 15).

### *Cultures of Mononuclear Blood Cells from Man and Rat with PHA*

In both human and rat blast cells some nuclear projections were occasionally observed. Some differences could be noticed in comparison with the findings described above. In blasts derived from human blood cells nuclear projections were obvious. However, they did not alter the nuclear outline as markedly as in leukaemic cells (Fig. 16, 17). In addition, the width of the inner perinuclear space was much more commonly irregular (Fig. 16); the area of cytoplasm surrounded by the nuclear projection appeared floccular, very poor or devoid of organelles (Fig. 16, 17). In rat blasts intermediate patterns were seen; the area surrounded by these projections was as rich in organelles as the outer cytoplasm (Fig. 18).

### DISCUSSION

The nuclear projections described in the present series are similar to those previously observed by many authors. A morphological observation consistently recorded in this work was the presence of a row of dense granules in the middle osmiophilic layer of the nuclear projections. This finding had not been described by other authors although it can be occasionally seen in their figures.

In the present material the projections were often situated in front of the Golgi area. The cytoplasm circumscribed by them was rich in ribosomes, vesicles, and dense bodies. These features might suggest highly developed interactions between nucleus and cytoplasm at the site of the projections. In fact, there is a more extensive contact surface between nucleus and cytoplasm at this level. Also Törő and Oláh (1966) consider the nuclear projections of thymocytes as related with the transference of nuclear material to the " protoplasm " but it is to determine whether this phenomenon is linked with the acquisition of immunological competence or whether the nuclear projections are associated " with the development of the polymorphic nucleus at mitosis. " In Anderson's leukaemia cases these nuclear changes were observed in myeloid cells, Huehns *et al.* (1964) found them in neutrophils, and in our cases of Hodgkin's disease they were present in a granulocyte and in Reed-Sternberg cells; thus they could not be specifically linked with transference of immunological information and they are not exclusive of lymphoid cells. Our finding suggest that they can be observed in cases of leukaemia when searched for. Anderson (1965, 1966) thinks that these features are perhaps more common following chemotherapy and in fact our cases of leukaemia were under treatment; nevertheless, the nuclear projections were present in an untreated case of leukaemia mentioned by Anderson (1966).

In the Reed–Sternberg cells nuclear projections are not as characteristic as in the leukaemic cells. Our findings are possibly comparable with the invaginations of the nuclear membrane discussed by Bernhard and Leplus (1964). These authors emphasized the frequent contact between the nucleoli and these clefts containing cytoplasm, and wondered whether the nucleolo-cytoplasmic exchange could be favoured by them.

In the *in vitro* studies described in the present work, nuclear projections were found in both human and rat blood mononuclear cells. As far as we know, this finding had not been recorded by others (Inman and Cooper, 1963; Tanaka *et al.*, 1963; Elves *et al.*, 1964; Martines, Musacci and Ricci, 1964; Mollo, Stramignoni and

---

EXPLANATION OF PLATES

FIG. 1.—Leukaemia, Case 1 (lymph node): a leukaemic cell (lc), shows indented nucleus, swollen mitochondria (m), and a nuclear projection (arrow). rc = reticular cell; mc = macrophagic cell; lyc = lymphoid cell. (TLR 147-455/66). ×9,750.

FIG. 2.—Leukaemia, Case 1 (lymph node): a nuclear projection surrounds a cytoplasmic area with a large vacuole (v). 1, 2 = external membranes of the nuclear envelope; (respectively inside and outside the nuclear projection); 3, 4 = Perinuclear space, (respectively outside and inside the nuclear projection); 5 = granular layer; 6 = inner membranes of the nuclear envelope. (TLR 147–621/66). ×72,500.

FIG. 3.—Leukaemia, Case 1 (blood): multiple nuclear projections. The arrows indicate the perinuclear space inside the nuclear projections; dense bodies are recognizable between cytoplasmic islands. (SA 149–713/66). ×41,000.

FIG. 4.—Leukaemia, Case 1 (blood): multiple nuclear projections: one of them surrounds a swollen mitochondrion (m). (SA 149–709/66). ×26,500.

FIG. 5.—Leukaemia, Case 1 (lymph node): nuclear projection surrounding a cytoplasmic area (c) with vacuoles (v), and very numerous ribosomes. Dense background. (TLR 147–683/66). ×28,500.

FIG. 6.—Leukaemia, Case 1 (blood): a racket-shaped nuclear projection surrounding a cytoplasmic area which is rich of vacuoles (v). (SA 149–627/66). ×35,500.

FIG. 7.—Leukaemia, Case 1 (blood): nuclear projection surrounding a cytoplasmic area containing two mitochondria (m). (SA 149–715/66). ×26,500.

FIG. 8.—Leukaemia, Case 1 (lymph node): ring-shaped nuclear projection (np), cut of the nucleus, surrounding a cytoplasmic area filled with ribosomes (c) and placed next to the Golgi structures (G). (TLR 147–667/66). ×40,500.

FIG. 9.—Leukaemia, Case 1 (lymph node): mitochondrion (m) closely adjacent to a nuclear projection, the latter surrounds a cytoplasmic area filled with ribosomes (c). (TLR 147–619/66). ×38,000.

FIG. 10.—Leukaemia, Case 1 (lymph node): on the right, a complex nuclear projection (np1); on the left, a nuclear projection (np2) cut next to the nucleus. (TLR 147–686/66). ×33,000

FIG. 11.—Leukaemia, Case 1 (lymph node): a nuclear projection (np) facing the Golgi apparatus (G); c = centriole; mt = microtubule. (TLR 147–623/66). ×33,000.

FIG. 12.—Leukaemia, Case 2 (blood): leukaemic cell with a pale nucleus showing a nuclear projection (np). (SA 172–1081/66). ×9,500.

FIG. 13.—Hodgkin's disease, Case 4 (lymph node): a neutrophil granulocyte, with some nuclear lobes (N), one of which shows a nuclear projection (np). (TLR 109–1175/66). ×15,500.

FIG. 14.—Hodgkin's disease Case 5 (lymph node): Reed-Sternberg cell, with large cytoplasm rich of small mitochondria; the lobulated nucleus shows several nuclear blebs containing granular material (b), and a nuclear projection (in the inset). (TLR 144–1331/66). ×5,500.

FIG. 15.—Hodgkin's disease, Case 5 (lymph node): detail of the marked area in Fig. 14: the nuclear projection shows an outer perinuclear space (ops) and an inner perinuclear space (ips) of the nuclear envelope, which separates the nucleus from the invaginated cytoplasm. (TLR 144–1335/66). ×29,500.

FIG. 16.—Culture from human mononuclear blood cells with PHA: a nuclear projection (np) in a blast cell: the outer perinuclear space (ops) is wide and the inner perinuclear space (ips) surrounds some islands of cytoplasm (c), which appears floccular. (BL 34–651/66). ×36,500.

FIG. 17.—Culture from human mononuclear blood cells with PHA: a nuclear projection (np) in a blast cell; an area of finely granular cytoplasm (c) is surrounded by it. (BL 33–422/65). ×29,500.

FIG. 18.—Culture from rat mononuclear blood cells with PHA: a nuclear projection (np) in a blast cell: the surrounded cytoplasm (c) shows the same patterns of the outer cytoplasm. (BLR 151–935/66). ×31,500.

1
lyc
lyc
rc
m
m
lc
mc
2
2
3
5
4
6
1
v
3
4
m

Mollo and Stramignoni.

Mollo and Stramignoni.

Mollo and Stramignoni.

Volante, 1966). It is interesting to notice that these cells are stimulated and actively proliferating. In our material some differences concerning nuclear projections between leukaemic cells and human blast PHA cells can perhaps be explained by the different fixation. In fact the rat PHA blast cells (fixed in the same manner as the leukaemic cells) showed nuclear projections similar to those of leukaemic cells.

A working hypothesis suggested by the data hitherto collected is that nuclear projections are linked with the increase of proliferation rate or metabolic activity of the cells; in any case it can be assumed that these features are dynamic nuclear changes.

SUMMARY

Peculiar nuclear projections similar to those found by Epstein *et al.* in Burkitt lymphoma cells are described in blood and lymph node cells of leukaemia and Hodgkin's disease cases as well as in lymphocytes treated *in vitro* with phytohaemagglutinin. The significance of these findings is briefly discussed.

### REFERENCES

Achong, B. G. and Epstein, M. A.—(1966) *J. natn. Cancer Inst.*, **36**, 877.
Anderson, D. R.—(1965) 'Discussion on the paper by Epstein, Barr and Achong', 'Methodological approaches to the study of leukemias'. Edited by V. Defendi, Wistar Inst. Monogr. No. 4, Discussion p. 81.
Anderson, D. R.—(1966) *J. Ultrastruct. Res.*, Suppl. 9.
Bernhard, W. and Leplus, R.—(1964) 'Fine structure of the normal and malignant human lymph node'. Oxford (Pergamon Press); Paris. (Gauthier-Villars); New York (Macmillan).
Cheville, N. F.—(1966) quoted by Achong and Epstein (1966).
Dorfman, R. F.—(1966) *Lancet*, ii 909.
Elves, M. W., Gough, J., Chapman, J. A. and Israëls, M. C. G.—(1964) *Lancet*, i, 306.
Epstein, M. A. and Achong, B. G.—(1965) *J. natn. Cancer Inst.*, **34**, 241.
Epstein, M. A., Barr, Y. M. and Achong, B. G.—(1965*a*) *Br. J. Cancer*, **19**, 108.—(1965*b*) 'Studies with Burkitt's lymphoma', 'Methodological approaches to the study of leukemias'. Edited by V. Defendi, Wistar Inst. Monogr. No. 4, p. 69.—(1966) *Br. J. Cancer*, **20**, 475.
Huehns, E. R., Lutzner, M. and Hecht, F.—(1964) *Lancet*, i, 589.
Inman, D. R. and Cooper, E. H.—(1963) *J. Cell Biol.*, **19**, 441.
Luft, J. H.—(1961) *J. biophys. biochem. Cytol.*, **9**, 409.
Martines, G., Musacci, G. and Ricci, N.—(1964) *Riv. Patol. Clin. Sper.*, **5**, 167.
Millonig, G.—(1962) 'Further observations on a phosphate buffer for osmium solutions in fixation, Electron Microscopy (*Proc. 5th int. Congr. Electron Microscopy*). New York (Academic Press). Vol. 2, P-8.
Mollo, F., Stramignoni, A. and Volante, G.—(1966) *Pathologica*, **58**, 23.
Palade, G. E.—(1952) *J. exp. Med.*, **95**, 285.
Papadimitriou, J. M.—(1966) *Proc. Soc. exp. Biol. Med.*, **121**, 93.
Parker, J. W., Wakasa, H. and Lukes, R. J.—(1967) *Lancet*, i, 214.
Reynolds, E. S.—(1963) *J. Cell Biol.*, **17**, 208.
Sabatini, D. D., Miller, F. and Barrnett, R. J.—(1964) *J. Histochem. Cytochem.*, **12**, 57.
Sebuwufu, P. H.—(1966) *Nature, Lond.*, **212**, 1382.
Tanaka, Y., Epstein, L. B., Brecher, G. and Stohlman, F. Jr.—(1963) *Blood*, **22**, 614.
Törö, I. and Oláh, I.—(1966) *Nature, Lond.*, **212**, 315.
Volante, G., Bussolati, G. and Stramignoni, A.—(1966) *Pathologica*, **58**, 1.